# Perceived Everyday Age Discrimination and Depressive Symptoms:The Moderating Effect of Social Environment

**DOI:** 10.1093/geroni/igaa057.3224

**Published:** 2020-12-16

**Authors:** Min Kyoung Park, Christine Mair

**Affiliations:** University of Maryland, Baltimore County, Baltimore, Maryland, United States

## Abstract

Approximately 30% of men and women in the United States have experienced age discrimination (Rippon, Zaninotto, & Steptoe, 2015). Experiencing age discrimination may lead to increased risk of depressive symptoms among older adults. Although positive social environments are known to buffer depressive symptoms, it is unknown to what extent a positive social environment may buffer the association between age discrimination and depressive symptoms for older adults in the US. The purpose of this study is to examine the association between perceived age discrimination and depressive symptoms among older adults, and to explore whether this association varies by two aspects of the social environment: social support and neighborhood environment. We explore this topic with data on 5,439 adults aged 50 and older in a sample drawn from the Psychosocial Module of the Health and Retirement Study (HRS, 2016 wave). Our results show a clear association between age discrimination and increased risk of depressive symptoms, net of a range of covariates. Older adults who receive more positive social support and rate their neighborhood environment more positively also report lower depressive symptoms. Finally, we find statistically significant interactions between age discrimination and both measures of the social environment, which suggest that social support and a positive neighborhood environment may buffer the negative impact of age discrimination on depressive symptoms. We discuss these findings in light of the prevalence of age discrimination in the US and cross-nationally, and consider potential mechanisms for improving the social environment of older adults, particularly in the post-COVID era.

